# A large-scale study and six-month follow-up of an intervention to reduce causal illusions in high school students

**DOI:** 10.1098/rsos.240846

**Published:** 2024-08-21

**Authors:** Naroa Martínez, Helena Matute, Fernando Blanco, Itxaso Barberia

**Affiliations:** ^1^ Departamento de Psicología, Universidad de Deusto, Bilbao, Spain; ^2^ Departamento de Psicología Social, Universidad de Granada, Granada, Spain; ^3^ Grup de Recerca en Cognició i Llenguatge (GRECIL), Departament de Cognició, Desenvolupament i Psicologia de l’Educació, Secció de Processos Cognitius, Institut de Neurociències, Universitat de Barcelona, Barcelona, Spain

**Keywords:** cognitive bias, science education, large-scale study, follow-up, causal illusion, debiasing, educational intervention

## Abstract

Causal illusions consist of believing that there is a causal relationship between events that are actually unrelated. This bias is associated with pseudoscience, stereotypes and other unjustified beliefs. Thus, it seems important to develop educational interventions to reduce them. To our knowledge, the only debiasing intervention designed to be used at schools was developed by Barberia *et al.* (Barberia *et al.* 2013 *PLoS One* **8**, e71303 (doi:10.1371/journal.pone.0071303)), focusing on base rates, control conditions and confounding variables. Their assessment used an active causal illusion task where participants could manipulate the candidate cause. The intervention reduced causal illusions in adolescents but was only tested in a small experimental project. The present research evaluated it in a large-scale project through a collaboration with the Spanish Foundation for Science and Technology (FECYT), and was conducted in schools to make it ecologically valid. It included a pilot study (*n* = 287), a large-scale implementation (*n* = 1668; 40 schools) and a six-month follow-up (*n* = 353). Results showed medium-to-large and long-lasting effects on the reduction of causal illusions. To our knowledge, this is the first research showing the efficacy and long-term effects of a debiasing intervention against causal illusions that can be used on a large scale through the educational system.

## 1. Introduction

Causal learning is the cognitive process of inferring causal relationships from available information. This ability to infer causal relationships in our environment has conferred important survival advantages to both humans and other animals since it allows us to anticipate changes and adjust our behaviour accordingly. However, our ability to detect causal patterns is not error-free. In some cases, it has been shown that individuals can erroneously infer a cause–effect relationship between events that are actually unrelated (see [1–3] for reviews). This cognitive bias is known as causality bias or causal illusion.

Causal illusions often lead to suboptimal decisions and can produce undesirable outcomes that underlie many social issues. For example, they have been associated with social stereotypes [4–6], ideological extremism [7,8], epistemically unwarranted beliefs such as paranormal [9], superstitious [10] and pseudoscientific beliefs [11–14], and the use of alternative and complementary medicine [15,16], among others.

There is evidence that not only adults but also children can show causal illusions [17]. In fact, children and adolescents might be especially vulnerable to causal illusion as they lack the basic cognitive skills and background knowledge exhibited by adults [18,19], which are important characteristics involved in causal judgement.

In the face of these threats to human well-being that are associated with causal illusions and other cognitive biases, the design of debiasing methods represents a major goal of modern psychology [20]. These debiasing methods aim to eliminate or at least diminish the intensity or frequency of cognitive biases, and can rest on different strategies or principles (see §4). A prominent strategy for debiasing is the implementation of educational interventions [21,22] to be used, ideally, as early as possible in life. At the moment, there is little research examining the effects of debiasing methods to reduce the causal illusion specifically. In particular, we do not know of any educational intervention to debias people against causal illusions other than the seminal one published by Barberia *et al.* [23] with adolescents, and a related one conducted with adults by Barberia *et al.* [24], Martínez *et al.* [25] and Rodríguez-Ferreiro *et al*. [26].

Next, we describe the study conducted by Barberia *et al.* [23] because it is the only study we are aware of that aimed to reduce causal illusions in adolescents, which is the target sample in the current study. Barberia *et al.* [23] used a convenience sample of 62 students who were randomly assigned to either the intervention or the control group. The intervention group received the intervention before the assessment of causal illusions, whereas in the control group, the assessment took place in the absence of target intervention. The intervention took the form of a critical thinking workshop especially designed for adolescents, and was delivered as part of a larger set of activities within a technology and robotics summer camp. This brief educational intervention, lasting for approximately 80 min, focused on training participants in base rate, experimental control conditions and confounding variables, as an appropriate approach for everyday causal inference. The intervention comprised two phases that were conducted face-to-face: a bias-induction staging phase (where participants were asked to experience the purported benefits of a bogus product in improving cognitive and physical abilities) followed by a training phase (where participants were revealed that the product did not have the claimed benefits and were trained in the design of experiments with appropriate control conditions for judging whether a product or a treatment is effective). The effectiveness of the intervention was assessed using a standard computerized contingency learning task (e.g. [27]). In this task, participants were required to imagine they were doctors evaluating the influence of a drug (i.e. a potential cause) on the recovery from a disease (i.e. the outcome) in a group of patients. They observed a number of patients (i.e. trials), and for each of them, they could decide whether to administer the drug or not, and they received feedback regarding the subsequent recovery of the patient. After all trials were completed, participants were asked to give a causal judgement indicating the degree to which they considered the drug to be effective against the disease.

The contingency learning task in Barberia *et al.* [23] included a null contingency condition (in which the probability of healing was high but was identical regardless of whether or not the drug was administered) and a positive contingency condition (in which the probability of healing increased noticeably when the drug was administered). Therefore, if participants erroneously judged that there was a causal relationship between the two events in the null contingency condition (i.e. if they believed that the drug was responsible for the cure of the patients), this was interpreted as a causal illusion, while the same judgement was considered an appropriate response in the positive contingency condition. The results showed that the intervention group developed a causal illusion significantly lower than that of the control group in the null contingency condition, while the positive contingency condition generated accurate causal judgements in both groups. The authors attributed the intervention’s direct effect on causal judgements to the success in prompting participants to focus more on control information (the probability of healing when the drug was not administered). Moreover, the reduction in causal illusions produced by the intervention appeared to be partially mediated by a change in the behaviour of the participants. Specifically, participants in the intervention group administered the drug to fewer patients, which might have facilitated their observation of patients who took the drug and patients who did not take it, a condition associated with less intense causal illusions (see [27]). These results suggest that the intervention influenced causal judgements both by promoting greater attention to the control information and by changing participants’ behaviour so that they administered the drug in a more balanced manner. This is consistent with the content of the intervention, which emphasized the importance of using proper controls when judging the effectiveness of a product, which in this case implied sampling information from patients who did not receive the drug as well as from those who did so that the two conditions could be compared.

In sum, the intervention developed by Barberia *et al*. [23] seems to be a promising approach for reducing causal illusions by helping adolescents understand the need for critical and scientific thinking when judging the efficacy of a product or treatment and the only one known for this purpose in this age group. However, Barberia *et al*.’s [23] intervention had some limitations. First, the study was a small-scale experimental project conducted with a small convenience sample, with potential selection bias issues (because the intervention was offered as a workshop to adolescents who had already shown an interest in technology and robotics). Moreover, the intervention did not include any follow-up measures in order to ascertain whether the immediate improvement would have the intended enduring protective effect against causal illusions.

Thus, the aim of the current research was to replicate these results in a large-scale project while improving the ecological validity (by conducting the intervention in the conditions of regular schools) and including a follow-up assessment of the efficacy of the intervention. Therefore, in this article, we report a pilot study, a subsequent large-scale study and, finally, a follow-up study conducted six months later. The research was carried out in collaboration with the Spanish Foundation for Science and Technology (FECYT). The data and materials for this research are publicly available at the Open Science Framework [28].

## 2. Studies 1 and 2: a pilot study followed by a large-scale study

### 2.1. Participants

A total of 287 high school students from grades 8 and 9 took part in the pilot study (mean age 13.60, s.d. = 0.62; 139 females, 147 males, one person did not disclose her/his gender). Participants were randomly assigned to one of the two groups: the intervention group included 151 participants, and the control group included 136 participants. The pilot study was conducted in three schools (one public and two partially subsidized) in three different regions of Spain (Cataluña, Comunidad de Madrid and País Vasco). All schools were located in large urban areas (over 50 000 inhabitants).

A total of 1668 high school students from grades 8 to 10 took part in the subsequent large-scale implementation (mean age 14.19, s.d. = 0.95; 839 females, 772 males, 57 self-identified as ‘other’). Participants were assigned to one of the two groups: the intervention group included 841 participants and the control group included 827 participants.

The large-scale study was conducted in 40 schools (20 public, 16 partially subsidized, 4 private) across four different regions of Spain: Andalucía, Castilla-La Mancha, Castilla y León and Comunidad de Madrid. These schools were located in 13 large urban areas (over 50 000 inhabitants) and seven rural areas (under 12 000 inhabitants). Therefore, there was sufficient diversity in terms of the type of school and geographical location. Among those schools that offered at least three class groups for the same grade, we invited only two of the groups to participate in this study, so that the remaining students would serve as a control group for the follow-up study that would be carried out six months later (see §3).

### 2.2. Design

In the pilot study, the assignment of participants to the different conditions was random. Note that in the case of the large-scale study, this fully random assignment was not possible due to the restrictions of the COVID pandemic, where bubble groups could not be modified in the schools. Because of this, the groups in the large-scale study coincided with the pre-existing school class groups and were then randomly assigned as either the intervention or control groups. The design, predictions and analyses of the pilot study were pre-registered in Aspredicted.org (available at https://aspredicted.org/48ax8.pdf). The large-scale study was not pre-registered but it was a replication of the pilot study.

In both studies, the intervention group received the complete intervention before the assessment, while the control group did not. During that time, the control group participated in a science outreach workshop (on nanotechnology in the pilot study, and on astronomy and space travel in the large-scale study) in order to ensure that an equal time was spent in a tutorized extracurricular activity external to their own school class just before assessment. The duration of the workshop was always the same as that of the intervention. Importantly, these workshops included scientific content but, unlike our intervention, they lacked an explanation of scientific methods and experimental control specifically.

Additionally, due to ethical considerations, the intervention was also provided in the control group, but only after the assessment was completed, so that it would not interfere with the evaluation of the effectiveness of the intervention. For the same reason, the control workshop was also offered to the intervention group once the study was completed. Finally, the two groups were carefully debriefed to make sure that they correctly understood the study goals.

### 2.3. Procedure

The pilot study was conducted between October and December 2019, and the large-scale implementation between October and December 2021. Both studies were conducted during regular class time and lasted about 90 min for each group. The activity was framed in the context of a school project led by Fundación Española para la Ciencia y la Tecnología (FECYT), whose purpose was to train high school students in scientific thinking and improve their ability to think critically. Two of the authors (N.M. and F.B.) conducted the intervention in the pilot study, while two instructors hired by FECYT and trained by the researchers conducted the intervention in the large-scale study. Two additional instructors conducted the science outreach workshops, both in the pilot study and in the large-scale study. The instructors met the following requirements: they held a university degree in natural sciences and they had at least one year of experience in science outreach and leading groups of different profiles and educational levels. In both studies, for the assessment phase, each participant used a computer, tablet or smartphone connected to the Internet and was encouraged to work individually. Although some schools differed in the chosen device to access the study, these differences did not affect the statistical results.

#### 2.3.1. Intervention

The intervention was conducted face-to-face and was a conceptual replication of Barberia *et al*. [23], with some minor adaptations that will be described in this section. As in Barberia *et al*. [23], the intervention included two phases and they were completed in a single session. The first phase involved a bias induction and the second one was a training phase.

##### 2.3.1.1. Bias induction phase

One of the main problems when developing debiasing techniques is that people usually think that they are free of biases (i.e. blind spot bias, see [29,30]), and thus it is difficult to correct a problem that they believe is a problem of other people, not theirs. Thus, the main purpose of this phase was to show them that they are vulnerable to causal illusions and their potential consequences so that they would be more motivated and interested in paying attention and learning how to correct this problem during the subsequent training phase. Although previous research with adults has shown that the bias induction phase is not always necessary [25], we thought it is important to maintain it when working with adolescents because it seems to increase motivation and engagement, students enjoy it, and the schools (as well as the students and their legal guardians) are more willing to participate in the study, and value it more when this phase is included. Indeed, in the absence of this phase, the intervention would resemble a regular class on scientific methods and experimental control of variables.

Thus, the induction phase was conducted with the aim of generating a biased judgement about the effectiveness of a target product among the participants, by using techniques that are common in advertising and pseudoscience. The target product was a metal ring (replacing the small piece of ferrite used by Barberia *et al.* [23]) that participants were asked to wear on their fingers. They were told that the product was made of a new material recently developed in a top research laboratory, which endowed it with special properties. It was explained to them that when the product contacted the skin, it increased the physical and cognitive capacity of the wearer. Following the strategy commonly used in pseudoscience, a hyper-technical explanation of the product was offered [7,31].

In addition, a series of exercises were carried out to further persuade participants into believing that those who used the ring improved their physical and cognitive capacities (note that, in order to keep the duration of the intervention brief, we reduced the number of exercises compared with the original intervention by Barberia *et al*. [23]). Importantly, all exercises used poor control conditions against which participants could compare their performance or did not include a control condition at all. First, participants performed two simple cognitive tasks (i.e. solving a maze and a memory game), always while wearing the metal ring, that is without testing a control condition. To increase their perception that the product was effective, participants were told that individuals in previous tests reported feeling that they had performed the tasks particularly well when using the ring. Second, participants engaged in two physical exercises (stability and flexibility), similar to those advertised by companies trying to show how some popular products improve sports performance, such as the power balance bracelet, which has been shown not to work [32]. Specifically, we asked two volunteers to perform each physical exercise on two successive occasions, in front of the class, always in the same order: first, without wearing the ring and, immediately afterwards, while wearing it. We asked the rest of the participants to observe the volunteers carefully. They could compare their performance with and without the product this time, but its effect was confounded by the possible influence of uncontrolled variables, such as the effect of practice and warm-up. As in the cognitive tasks, the experimenters made comments to fuel the perception of the effectiveness of the ring, and the volunteers’ performance was reinforced with enthusiasm even when there was no visible improvement. Before proceeding to the next phase, a manipulation check for this bias induction phase was included. Specifically, the intervention group was asked to evaluate the effectiveness of the ring on a scale with four response alternatives, i.e. ‘It does not work’ (0), ‘It works a little’ (1), ‘It works quite well’ (2) and ‘It works very well’ (3). Additionally, the intervention group was also asked to estimate how much they thought the ring cost on a scale with four response alternatives in the pilot study, i.e. ‘less than 10 euros’ (0), ‘between 10 and 50 euros’ (1), ‘between 100 and 200 euros’ (2) and ‘more than 200 euros’ (3), and a scale with six response alternatives in the large-scale study, i.e. ‘less than 10 euros’ (0), ‘between 10 and 25 euros’ (1), ‘between 25 and 50 euros’ (2), ‘between 50 and 100 euros’ (3), ‘between 100 and 200 euros’ (4) and ‘more than 200 euros’ (5). Note that these questions were not included in the original intervention by Barberia *et al.* [23].

##### 2.3.1.2. Training phase

This phase started by revealing the ineffectiveness of the ring. Then, an explanation was provided about the mistakes that were made when testing the ring, what could have been done to detect the fraud and how to apply the experimental method and adequate control conditions when testing causal links. Specifically, we instructed participants on the importance of control conditions in order to evaluate a causal relationship. First, using the example of an alleged remedy against a common cold, they were educated on the necessity of comparing the probability of recovery from the cold when using the remedy with the probability of recovering spontaneously with no intake of the remedy. This comparison, and not simply the fact of recovery being very likely when taking the remedy, was presented as the key element to consider when evaluating its effectiveness. Moreover, it was noted that, in our daily life, if spontaneous recovery from a disease, such as a cold, is very likely, then the chances that the intake of any presumed remedy will coincide with an improvement of the health condition are very high. And that these coincidences might encourage people to believe that the remedy is working, and therefore, to continue consuming it, even if it is not effective at all.

Second, through an example of a plant fertilizer, they were further introduced to the idea of appropriate versus inappropriate control conditions. They were asked to imagine that the effectiveness of the fertilizer was tested by administering it to a plantation in Bilbao (a Spanish city with rainy weather) and comparing the growth of this plantation with that of a plantation to which no fertilizer was applied and was located in the Sahara Desert. The example was used to bring to attention the fact that, even if plants in the first farm grew bigger, this would not necessarily mean that the fertilizer was effective, given that the effect of other relevant variables (i.e. the weather) was not controlled across plantations.

These two examples served to analyse the deficiencies in the evaluation of the ring in the previous induction phase (i.e. no control condition when performing the initial cognitive exercises and inadequate control of the effect of practice and warming-up when performing the subsequent physical exercises).

### 2.3.2. Assessment

After the intervention, the assessment of the effectiveness of the intervention was conducted using a standard contingency learning task, that was very similar to that used by Barberia *et al*. [23]. Participants completed the task on a computer and they were asked to imagine being a medical doctor. They were told that their task was to determine whether a fictitious drug was effective in providing relief to patients suffering from a fictitious disease. The records of 40 fictitious patients suffering from the disease were presented sequentially, one per trial. In each trial, participants decided whether they wanted to administer the drug to the patient or not. Then, participants observed whether that patient felt relieved. After all 40 trials were completed, participants were asked to evaluate the effectiveness of the drug on a scale ranging from 0 to 100 (with the labels of ‘not effective at all’, ‘moderately effective’ and ‘totally effective’ for 0, 50 and 100 values, respectively). Causal judgements on this scale along with the mean P(Cause) in the contingency learning task were our main dependent variables. P(Cause) refers to the proportion of trials in which the participants administered the drug (i.e. the potential cause of recovery) to the fictitious patients. As shown by Barberia *et al*. [23], participants tend to show a high P(Cause) by default, so reducing P(Cause) to a value close to 0.50 might be a key factor in the effectiveness of the intervention in reducing causal illusions. Specifically, since participants during the intervention are instructed on the importance of control conditions when evaluating a causal relationship, this should translate into a less biased behaviour in the contingency learning task so that they would introduce the potential cause, ideally, in 50% of the trials. This should produce a more balanced exposition of both trials in which the candidate cause is present and absent, as compared with control participants, who would exhibit the default tendency to often administer the drug to the patients and, therefore, expose themselves to biased information (i.e. a higher number of cause-present trials).

All participants were presented with a null contingency and a positive contingency problem. In the null contingency problem, the probability of recovery of the patients did not change when administering the drug (the probability was always 0.75), whereas in the positive contingency problem, the probability increased from 0.125 to 0.75 when administering the drug. The high rate of recovery in the null contingency problem was included in order to induce the development of strong causal illusions (e.g. [33]). Note that appropriate evaluations of the drugs would involve low causal judgements for the null contingency problem (the more a participant departs from the correct value of zero, the stronger the causal illusion he or she is showing) and medium-to-high causal judgements for the positive contingency one (in this case, the evidence suggests that the drug is effective). The order of presentation of the null and the positive contingency problems was randomly determined for each participant. In the first presented problem, the fictitious drug was called Batatrim and the fictitious disease was called Lindsay syndrome. In the second presented problem, the fictitious drug was called Dugetil and the fictitious disease was called Hamkaoman syndrome. Except for differences in the probabilities of recovery with and without the drug, the procedure was exactly the same in both problems.

The critical problem in which we aimed to observe the effect of our intervention was the null contingency problem. In this problem, we expected that participants exposed to the intervention would show a reduced causal illusion (i.e. lower causal judgements) compared with the control group. Moreover, we expected the intervention group to show a lower P(Cause) and we hypothesized that this variable would (at least partially) mediate the impact of the intervention on causal illusions. The positive contingency learning task was added in order to control for a potential confounding effect of scepticism: if the participants receiving the intervention became less likely to assume a causal relation not only in the null contingency scenario but also in the positive scenario, then we could assume that this was due to an increase in general scepticism rather than an improved accuracy in causal inference.

### 2.4. Results

The results from the two studies (pilot and large-scale intervention) will be reported together in this section, although they will be analysed separately.

#### 2.4.1. Manipulation check

First, we describe the results of the questions that were only asked to the intervention group after the bias induction phase. Table 1 reports the frequencies of the answer options to the first question that asked about the perceived effectiveness of the ring.

**Table 1.** Frequencies of the answers regarding the perceived effectiveness of the ring in the pilot and large-scale studies.

Perceived effectiveness	Pilot study (*n* = 95)	Large-scale study (*n* = 837)
0: ‘It does not work’	17 (17.9%)	82 (9.8%)
1: ‘It works a little’	25 (26.3%)	231 (27.6%)
2: ‘It works quite well’	45 (47.4%)	372 (44.4%)
3: ‘It works very well’	8 (8.4%)	152 (18.2%)

Note. The sample size of the pilot study corresponds to the experimental group from two of the three schools, as participants from one school did not answer these questions.

The mean rating for the perceived efficacy of the ring, on a scale from 0 to 3, was 1.47 (s.d. = 0.877) in the pilot study and 1.71 (s.d. = 0.875) in the large-scale study. These values fall between the labels ‘It works a little’ and ‘It works quite well’. In fact, as the table shows, ‘It works quite well’ was the most frequently selected label in both studies, and the vast majority of the sample (82% in the pilot study and 90% in the large-sample study) indicated that the ring worked at least to a little extent.

Then, participants in the intervention group were asked to guess the cost of the ring after the activities in the bias induction phase were carried out. The answers are summarized in table 2.

**Table 2.** Frequencies of the answers to the second question regarding the perceived cost of the ring in the pilot and large-scale studies. Note that the two studies used slightly different response scales.

Perceived cost	Pilot study (*n* = 95)
0: ‘less than 10 euros’	13 (13.7%)
1: ‘between 10 and 50 euros’	49 (51.6%)
2: ‘between 100 and 200 euros’	30 (31.6%)
3: ‘more than 200 euros’	3 (3.1%)

	Large-scale study (***n*** **= 837**)
0: ‘less than 10 euros’	117 (14.0%)
1: ‘between 10 and 25 euros’	240 (28.7%)
2: ‘between 25 and 50 euros’	184 (22.0%)
3: ‘between 50 and 100 euros’	131 (15.6%)
4: ‘between 100 and 200 euros’	109 (13.0%)
5: ‘more than 200 euros’	56 (6.7%)

Note. The sample size of the pilot study corresponds to the experimental group from two of the three schools, as participants from one school did not answer these questions.

The mean rating for the perceived cost of the ring, on a scale from 0 to 3, was 1.24 (s.d. = 0.72) in the pilot study. This value falls between the labels ‘between 10 and 50 euros’ and ‘between 50 and 200 euros’. In the large-scale study, the mean rating for the perceived cost of the ring (which was obtained on a scale from 0 to 5) was 2.05 (s.d. = 1.46). This value corresponds to the label ‘between 25 and 50 euros’. These results suggest that the bias induction phase worked as we had intended in the intervention group because in both studies the responses point to a perception that the ring works to some extent (and that it has a certain economic value).

#### 2.4.2. Assessment

As planned in the pre-registration, data from participants whose P(Cause) = 0, that is those who did not administer the drug in any trial during the contingency learning task, were excluded from the analyses. In the pilot study, this resulted in the exclusion of data from seven participants (six in the intervention group and one in the control group; final *n* = 280). In the large-scale implementation, this criterion excluded the data from eight participants (four from each of the two groups; final *n* = 1660).

**Figure 1.**
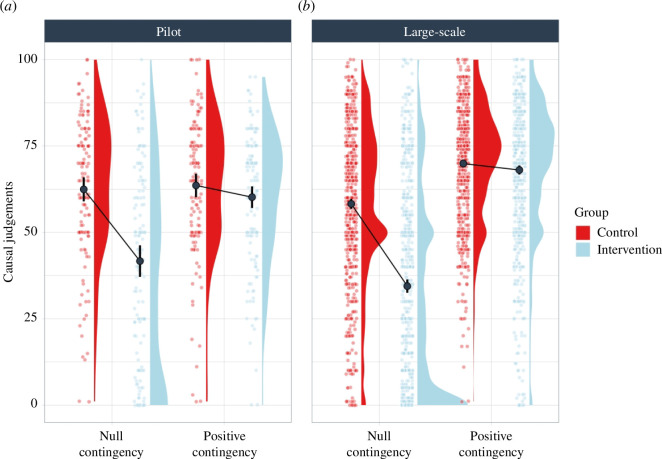
Causal judgements in the intervention and control groups in both contingency conditions (positive and null) and for the two studies (pilot study and large-scale study). Error bars depict 95% confidence intervals for the mean.

Figure 1 shows the mean causal judgements for each contingency condition in each of the intervention and control groups, both for the pilot (figure 1*a*) and for the large-scale implementation (figure 1*b*). As we expected, when the problem comprised a null contingency, the intervention group showed lower causal judgements (i.e. reduced causal illusion) compared with the control group. Conversely, in the positive contingency problem (where there was a genuine causal relation), no differences between the two groups seemed to emerge.

These observations were confirmed by two separate 2 × 2 mixed ANOVAs (group: intervention versus control; contingency: null versus positive) on the causal judgements, one for each study (pilot and large-scale). In both cases, there was a significant main effect of group: F_1,278_ = 34.50*, p *< 0.001, $\eta_{p}^{2}$ = 0.110 (pilot study) and F_1,1658_ = 281.82*, p *< 0.001, $\eta_{p}^{2}$ = 0.145 (large-scale study). Additionally, the main effect of contingency was significant: F_1, 278_ = 34.69*, p *< 0.001, $\eta_{p}^{2}$ = 0.111 (pilot study) and F_1,1658_ = 942.88*, p *< 0.001, $\eta_{p}^{2}$ = 0.363 (large-scale study). Finally, the group by contingency interaction was significant in both samples: F_1,278_ = 27.11*, p *< 0.001, $\eta_{p}^{2}$ =0.089 (pilot study) and F_1,1658_ = 221.93*, p *< 0.001, $\eta_{p}^{2}$ = 0.118 (large-scale study). The interactions were examined with non-parametric Mann–Whitney contrasts, due to the violation of the assumption of equal variances. Thus, when judging the null contingency problem, participants in the intervention group developed a weaker causal illusion than participants in the control group. This was observed in both the pilot study, *Z* = 5512, *p *< 0.001, *d* = 0.44, and in the large-scale study, *Z* = 177751.50, *p *< 0.001, *d* = 0.48. Meanwhile, the intervention and control groups showed similar causal judgements in the positive contingency condition: in the pilot study, *Z* = 8781, *p* = 0.137, *d* = 0.10 and in the large-scale study, *Z* = 337495, *p* = 0.477, *d* = 0.20. These results are consistent with our predictions and suggest that the intervention was successful in helping participants detect the absence of a causal relationship, particularly, in the condition where there was no evidence of it.^1^ At the same time, it did not prevent them from detecting the presence of a causal relationship in the condition where there was strong evidence of it.

**Figure 2.**
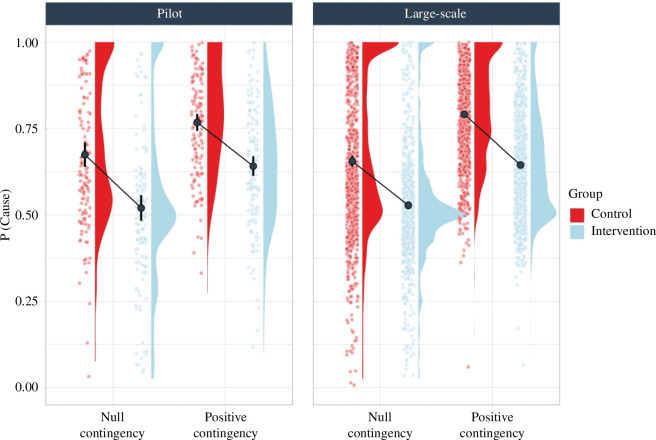
P(Cause) in the intervention and control groups in both contingency conditions (null and positive contingency), in the two studies (pilot study and large-scale study). Error bars depict 95% confidence intervals for the mean*.*

Regarding the behaviour of the participants during the contingency learning task, we analysed the proportion of patients to whom they administered the drug, P(Cause), in the critical null contingency condition. Our prediction was that participants in the control group would show a higher P(Cause) than those in the intervention group, who should display a more balanced proportion of patients taking the drug versus those not taking the drug. As reflected in figure 2, the results were consistent with our prediction, with the intervention group showing a lower P(Cause) than the control group, both in the pilot study, F_1,278_ = 35.3*, p *< 0.001, $\eta_{p}^{2}$*^^* = 0.113 and in the large-scale study, F_1,1658_ = 170.0*, p *< 0.001, $\eta_{p}^{2}$ = 0.093. Specifically, the control group administered the drug in 67.4% of the trials in the pilot study and in 65.5% of the trials in the large-scale study, while P(Cause) in the intervention group was closer to a balanced sampling: 52% of the trials in the pilot study and 52.8% of the trials in the large-scale implementation.

**Figure 3.**
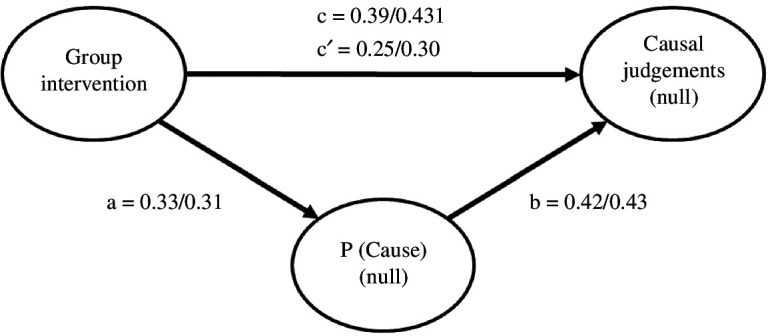
Mediational structure underlying the experimental manipulation in the null contingency condition in the pilot and large-scale studies. The total effect of the group on the causal judgements in the null contingency condition (path c) is divided into two components: one indirect effect (paths a and b) explained by the mediation of P(Cause), and one direct effect (path c′) of the intervention that remains significant after the indirect effect result has been discounted. The mediation analysis revealed a partial mediation, wherein the intervention affected the judgements both indirectly, via the P(Cause), and directly. The path values refer to the pilot/large-sale studies, respectively*.*

We also examined the role of the P(Cause) in mediating the influence of the intervention on causal illusions by conducting a mediation analysis using the ‘jAMM’ package for jamovi [34]. Figure 3 depicts the tested mediational structure.

We comment on the results of the pilot study first. The total effect of group (intervention versus control) on the causal judgements (β = 0.391, *p *< 0.001) was partially mediated by P(Cause): the indirect component was significant (β = 0.141, *p *< 0.001), as well as the remaining direct component (β = 0.250, *p* < 0.001). Given that the direct effect was significant after discounting the effect of P(Cause), the mediation was partial, as a part of the total effect is not mediated (i.e. the total effect is reduced by 36%). The results of the large-scale intervention were identical. The total effect of the intervention on the causal judgements was significant, β = 0.431, *p* < 0.001. This effect was decomposed on an indirect effect through P(Cause) that was significant, β = 0.133, *p* < 0.001 and a direct effect that remained significant, β = 0.298, *p* < 0.001 (the direct effect is reduced by 27% of the total effect). Again, this can be interpreted as a partial mediation of the effect of the intervention through P(Cause). Overall, these results suggest that the intervention impacted causal judgements directly by producing more realistic judgements but also indirectly by decreasing the participants’ tendency to favour cause-present trials.

## 3. Follow-up study

The follow-up study was conducted six months after the large-scale study described in previous sections, with the goal of examining the durability of the effect of the intervention. To this end, we combined participants who had taken part in the large-scale study with naive participants who had not taken part in any previous study.

### 3.1. Participants

A total of 353 high school students from grades 9 and 10 took part in this study (mean age 15.10, s.d. = 0.81; 192 females, 143 males, 16 self-identified as ‘other’, 2 did not disclose their gender). The follow-up study comprised two groups. The intervention group included 257 students who had participated in the large-scale study six months before. The control group included 96 additional participants who did not take part in any of the previous studies but were of similar age and attended the same school and course as the intervention group (see §3.2.).

The follow-up study was conducted in 10 of the 40 schools (nine public, one partially subsidized) from the previous large-scale study, corresponding to three different regions of Spain (Castilla-La Mancha, Castilla y León and Comunidad de Madrid). The schools were located in four large urban areas (over 50 000) and three rural areas (under 12 000 inhabitants).

### 3.2. Design

The follow-up study was conducted six months after the large-scale study. From the sample in the large-scale study, we chose the schools that offered at least three class groups of the same grade: two class groups that received the intervention in the previous large-scale study and at least one class group that did not receive any intervention, which would serve as our follow-up control group. This ensured that the intervention and control groups were comparable in this follow-up study. Note that the follow-up intervention group now combines participants who had already participated either in the intervention group or in the control group of the large-scale study, given that all of them had already completed the intervention phase by this moment (recall that, due to ethical considerations, the control group had also received the intervention, only that they received it once the study was completed). This follow-up study was pre-registered in Aspredicted.org (available at https://aspredicted.org/er76x.pdf).

### 3.3. Procedure

The study was conducted online during regular class time for about 45 min with each group, between April and June 2022. In each school, the teachers supervised the activity. This time, the procedure was identical in the two groups. It consisted of only an online assessment test using a contingency learning task, which was different from the one used in the previous studies.

The assessment consisted of a contingency learning task structurally analogous to that employed in the previous studies but with a very different cover story that involved learning about alien mutations [35], in order to test not only the long-lasting effects but also the generalizability of the intervention. Specifically, participants were asked to imagine themselves as scientists from the future working in a laboratory specialized in a species of evil aliens. Their task was to determine the effectiveness of a fictitious injection in causing a mutation that would render the aliens harmless to humans. Note that this futurist scenario parallels the medical scenario used in previous experiments, but instead of learning about the effectiveness of a drug to heal a disease, participants must now learn about the effectiveness of an injection to produce alien mutations. The purpose of this change in the cover story was to test the generalization of the effects of the intervention. That is, this change aimed to test whether participants would be able to apply what they had learned about variable control and experimental methods to correctly infer causal relationships in a completely new context. It should be noted that in the assessment phase of the large-scale study, we used a medical scenario, consistent with one of the examples that were explicitly provided during the intervention phase (a remedy for recovering from a cold). In contrast, in the follow-up assessment, we used an alien mutation scenario, which was absolutely different from the scenario presented in the assessment of the large-scale study, as well as from the examples used in the intervention six months before. If learning was effective, sufficiently general, and sufficiently perdurable, then it should transfer to this new task conducted six months later.

The two problems that were used in the null and the positive contingency problems in this follow-up study used different injections (i.e. candidate causes) and mutations (i.e. outcomes): in the first presented problem, Mutaprot was tested to produce the XG vulnerability, while in the second problem, the fictitious injection was called Protovin and the fictitious mutation was called ZY vulnerability. In addition, to streamline the task execution, the number of trials was reduced to 32, but we used the same probability of the outcome event in the presence and absence of the candidate cause event as in the previous studies. Thus, in both conditions, whenever the injection was administered, the probability that aliens mutated to be harmless was high (0.75). When the injection was not administered, the probability of the mutation remained equally high (0.75) in the null contingency condition, but low (0.125) in the positive contingency condition (therefore, in this latter condition, the mutation was more likely when the injection was administered than when it was not).

If the effect of the intervention persisted six months later, we expected the control group (who did not receive the educational intervention) to develop stronger causal illusions than the intervention group in the null contingency condition, while both groups would be equally accurate in detecting a positive contingency in the positive contingency condition.

### 3.4. Results

As in the previous studies, data from participants whose P(Cause) = 0, that is those who did not administer the drug in any trial during the contingency learning task, were not included in the analyses. Data from five participants (three in the intervention group and two in the control group) were excluded for this reason. Thus, the final *N* was 348, comprising 254 participants in the intervention group and 94 participants in the control group.

**Figure 4.**
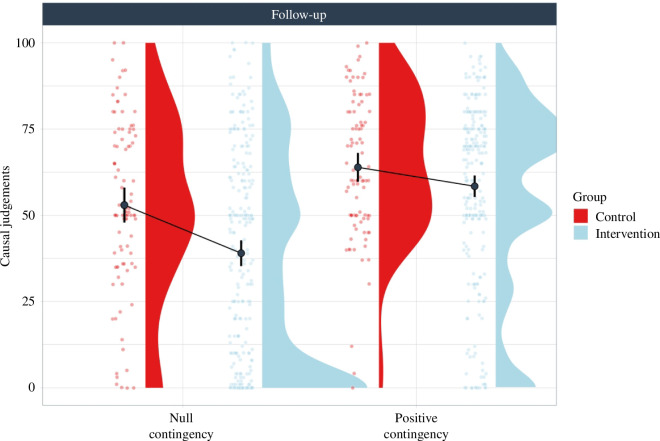
Causal judgements in the intervention and control groups in the two contingency conditions (null and positive) in the follow-up study. Error bars depict 95% confidence intervals for the mean*.*

Figure 4 shows the mean causal judgements for both groups and contingency conditions. The results observed six months after the intervention seem to parallel those observed in the immediate assessment in the pilot and large-scale studies. That is, while both groups provided high and similar causal judgements in the positive contingency condition, the intervention group provided lower causal judgements (i.e. showed a less intense causal illusion) than the control group in the null contingency condition.

These observations were confirmed by a 2 × 2 mixed ANOVA (group: control versus intervention; contingency: null versus positive) on causal judgements. This showed a significant main effect of group, F_1,346_ = 15.5*, p *< 0.001, $\eta_{p}^{2}$ = 0.043, a significant main effect of contingency, F_1,346_ = 54.63*, p *< 0.001, $\eta_{p}^{2}$ = 0.136, and a significant interaction between group and contingency, F_1,346_ = 4.25*, p* = 0.040, $\eta_{p}^{2}$ = 0.012. Post hoc comparisons (Mann–Whitney) revealed that participants in the intervention group developed a weaker causal illusion than participants in the control group in the null contingency condition, *Z* = 15 122, *p <* 0.001, *d* = 0.27, whereas in the positive contingency condition, the causal judgements did not differ significantly between groups, *Z* = 12 964, *p =* 0.218, *d* = 0.09. This suggests that, six months later, the intervention still allowed participants to detect the absence of a causal relationship in the condition where there was no evidence for it (in fact, the follow-up retains 51% of the original effect that was observed in the large-scale study) and at the same time, it did not prevent them from detecting the presence of a causal relationship in the condition where there was strong evidence for it.

Moreover, aligning with the results observed in the immediate assessment, six months after the intervention participants still produced a lower P(Cause), *M* = 0.602, s.d. = 0.22, than did control participants, *M* = 0.655, s.d. = 0.21, F_1,346_ = 4.12, *p* = 0.043, $\eta_{p}^{2}$ = 0.012, indicating that participants in the intervention group administered the injection less frequently (i.e. they behaved in a less biased manner) than those in the control group.

**Figure 5.**
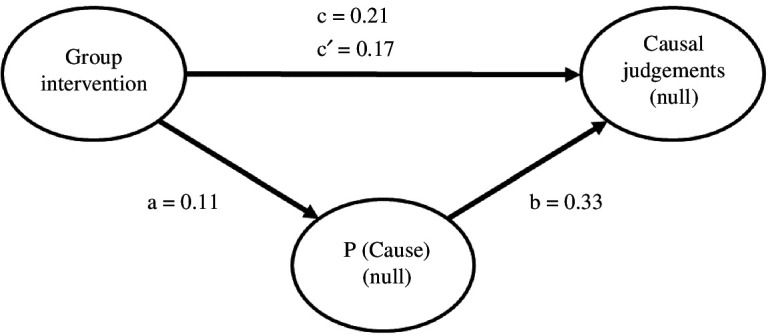
Mediational structure underlying the experimental manipulation in the null contingency condition (follow-up study). The total effect of the group on the causal judgements in the null contingency condition (path c) is divided into two components: one indirect effect (paths a and b) explained by the mediation of P(Cause), and one direct effect (path c′) of the intervention that remains significant after the indirect effect result has been discounted. Thus, the mediation analysis revealed a partial mediation, wherein the intervention affected the judgements both indirectly, via the P(Cause), and directly*.*

Finally, we also examined the role of the intervention on the judgements of causality in the null contingency condition by conducting a mediation analysis, as we did in the previous study. Figure 5 depicts the mediational structure underlying the experimental manipulation in the null contingency condition and the standardized path estimates.

The analyses revealed a significant total effect of the intervention on causal judgements, β = 0.210, *p *< 0.001. This total effect was decomposed into two paths. The indirect path through P(Cause) was marginally significant, β = 0.036, *p* = 0.051. The direct path, after partialling out the indirect effect, remained significant, β = 0.174, *p *< 0.001 (albeit reduced by 19% of the total effect). Overall, our results suggest that the intervention clearly reduced the illusion of causality even six months after the study, and it seems that P(Cause) was still mediating this effect. However, the evidence of this mediation after six months is not entirely clear, possibly because the direct effect of the intervention remains strong.

## 4. General discussion

In this article, we report a nationwide study consisting of a pilot study, a large-scale implementation and a follow-up assessment with the aim to replicate and extend the results of the intervention designed by Barberia *et al*. [23] to reduce causal illusions. The results robustly showed a medium to large effect of the educational intervention in reducing causal illusions among adolescents. In addition, and as we expected, the results indicated no significant differences between the intervention and control groups in their causal judgements when facing a positive contingency problem. This finding indicates that participants who received the intervention improved their ability to detect the absence of a causal relationship as compared with the control group, while still being able to detect the presence of a causal relationship when it existed. These results replicate the findings reported by Barberia *et al*. [23] and are consistent with the findings from similar interventions conducted with adults to reduce causal illusions [24,25]. Furthermore, they extend the results of these studies by testing a large-scale implementation of the intervention in high schools across Spain and, crucially, by incorporating the assessment of generalization and long-term effects. Considering the prevalence of weak or failed replications in psychology [36–38], this large-scale and long-term replication of previous empirical findings enhances our confidence in the efficacy of this type of debiasing intervention.

We also conducted mediation analyses to examine the role of P(Cause) in the relationship between the intervention and causal illusions. Consistent with Barberia *et al.* [23], our results revealed that P(Cause) partially mediated the immediate effect of the intervention on causal illusions. This partial mediation, where the direct effect persists, suggests that our intervention operates through two mechanisms during the contingency learning task: (i) by promoting a more balanced sampling of cause-present and cause-absent observations and (ii) by directing attention to the outcome occurrences when the candidate cause is absent. Regarding the first mechanism mentioned above, we observed that the tendency of the participants towards including the potential cause in many trials, that is the tendency to produce a high P(Cause), was consistently reduced in the intervention group as compared with the control group. This result indicates that participants in the intervention group understood the importance of experimental control and proper comparisons, thereby influencing their information sampling strategies. Thus, they generated more cause-absent observations than those in the control group. This allowed them to sample a more balanced and informative set of observations during the contingency learning task, leading to a more accurate estimation of causality. This can be seen as one potential mechanism for the success of the intervention. Consistent with these findings, previous experiments showed that the probability with which the potential cause is presented influences the development of causal illusions [39–41].

As for the second mechanism mentioned above, the intervention had a significant and direct effect on final causal judgements independent of the P(Cause) generated by the participants. Therefore, it seems likely that the intervention influenced causal judgements by highlighting the base rate of outcomes (e.g. what happens when no treatment is given) and encouraging participants to pay more attention to or give more weight to cause-absent information (see [42], which discusses the effect of base-rate instructions on causal illusion). This interpretation is supported by previous research conducted with adults, where debiasing interventions similar to ours have shown effectiveness in both active and passive contingency learning tasks [24–26]. Furthermore, our follow-up assessment extended the results of previous studies by showing that the effect of the intervention in reducing causal illusions was still strong after six months. We are not aware of any previous studies assessing the duration of the effect of a debiasing strategy like this one. Indeed, some researchers have noted that most debiasing strategies may not be robust enough to generate lasting effects and that most studies do not include a follow-up assessment through a longitudinal assessment [20,25]. This is important because studies conducted with adults suggest that erroneous beliefs (such as the causal illusion) may persist if not repeatedly and explicitly addressed [43,44]. In the present study conducted with high school students, we observed a robust and long-lasting effect of the intervention on the reduction of the causal illusion. Moreover, long-term benefits seemed attributable to the intervention producing more realistic causal judgements and also to changes in the behaviour, i.e. less-biased P(Cause) of the participants, although the results from the mediation analysis were not entirely clear due to the strong remaining direct effect. To interpret this absence of indirect effect in the follow-up, we can only speculate but, as discussed above, it is likely that the intervention works in two ways: directly and indirectly, through P(Cause). It seems possible that the indirect pathway became relatively more weakened with time (compared with the direct effect of the intervention). That is, participants who underwent the intervention would not reduce their P(Cause) to a great extent but still pay attention to cause-absent trials compared with controls.

Finally, as noted by some authors [25,45], it is important to test the possible generalization of the effect of the intervention to different problems and contexts, and it has been pointed out that debiasing strategies should show their effectiveness in multiple and diverse problems to be useful [20]. For this reason, the contingency learning task that we used in the follow-up study was different from the one used in the large-scale study in terms of the set-up and content. In this regard, our results showed an effect of the intervention on reducing causal illusions in a task and context that are not directly addressed in the intervention. Thus, the overall results do not only speak about the efficacy and duration of the intervention but also about the generalization to different contexts. The observation of this transfer of learning to other problems and contexts after a debiasing intervention is important, given the evidence, which suggests that critical thinking often fails to generalize to different situations and is disappointingly specific to the tasks explicitly addressed [46]. Further research should test the generalizability of the results to a wider range of tasks, provided that they share a common structure with ours: a potential candidate is the illusory correlation paradigm [5], which involves null contingencies but in a completely different domain that contains no specific reference to experimentation, science or scientific reasoning. Testing our intervention in such diverse contexts would help determine its broader applicability and effectiveness.

This work represents a successful collaboration with the national education system through the Spanish Foundation for Science and Technology and has significant implications. To the best of our knowledge, the present study is the first to show the efficacy of a large-scale and long-lasting debiasing intervention for causal illusions. The findings presented herein are an important contribution considering what is mentioned by Lilienfeld *et al.* [20, p. 391], ‘if researchers found debiasing to be efficacious and implemented it on a grand scale, it could prove to be scientific psychology’s most important contribution to reducing ideological extremism and both inter- and intragroup conflict’.

This study also contributes to the literature on debiasing strategies by shedding light on the robust effectiveness of a training procedure that promotes the role of teaching scientific methods in reducing biases [23]. Consistent with this view, recent research by Chow *et al*. [42] has also shown that even simple textual instructions on the scientific method can provide some protection against causal illusions. Thus, it seems that further exploring the potential of scientific teaching as a debiasing strategy should be a fruitful path that should contribute to reducing the illusion of causality and the problems associated with it. Other successful debiasing interventions include statistical reasoning, rules of formal logic and critical thinking, but there has been criticism based on the lack of theoretical coherence between different debiasing techniques, and on the mixed results in research on its effectiveness [47]. As for statistical reasoning, unless training is extensive, results often do not generalize to new problems or situations [48–50]. Similarly, training participants in the rules of formal logic has reported weak effects in preventing cognitive bias such as confirmation bias [51,52]. Research suggests that critical thinking skills are often domain-specific and rarely extend beyond the tasks in which they are taught [20,46,53].

In conclusion, our replication of a successful intervention carried out on a large-scale sample, in an ecological context but through controlled experimental design, and the addition of a generalized six-month follow-up assessment, as presented herein, is a promising avenue [45,54] that can build bridges between psychological science, policy-makers and the educational system, through successful collaborations for the public good. Indeed, our intervention has shown to be effective, generalizable and durable in reducing causal illusions, which are one of the best-known causes of pseudoscientific and other harmful beliefs and practices. Our research shows that this intervention could be easily applied in schools if there exists a good collaboration between scientists, policy-makers and the educational system.

## Data Availability

The raw data and materials for this study are available at [28].

## Footnotes

^1^
Note that in this task in which participants decide whether to introduce the potential cause or not, the contingency that participants actually expose themselves to can vary between individuals. If this individual contingency is controlled for in the analyses as a covariate, the conclusions hold the same in all the studies in this report. In the article, we report the analyses without covariates for the sake of simplicity.
